# Characteristics of chronic ulcer patients by gender and ulcer aetiology from a multidisciplinary wound centre

**DOI:** 10.1111/iwj.70012

**Published:** 2024-08-06

**Authors:** Katarina Kuikko, Teea Salmi, Heini Huhtala, Teija Kimpimäki

**Affiliations:** ^1^ Faculty of Medicine and Health Technology Tampere University Tampere Finland; ^2^ Department of Dermatology Tampere University Hospital Tampere Finland; ^3^ Faculty of Social Sciences Tampere University Tampere Finland

**Keywords:** comorbidity, diabetes mellitus, hypertension, leg ulcer, sex

## Abstract

Chronic ulcer patients form a heterogenous group of patients with various medical backgrounds. Cost‐effective targeted treatment necessitates more knowledge about specific features related to different subgroups of ulcer patients. Hence, this study aimed to characterize ulcer patients according to gender and ulcer aetiology. A total of 946 consecutively recorded chronic ulcer patients in the Tampere Wound Registry (TWR) were included and data were gathered from the TWR and patient medical records. Comparisons were made between males and females and patients with venous‐, arterial or mixed‐, diabetic foot‐, pressure‐ and atypical ulcers. Male patients were found to have diabetes, hypercholesterolemia and obesity significantly more often than females (59.2% vs. 39.6%; *p* < 0.001, 46.5% vs. 33.3%; *p* = 0.001, 42.7% vs. 35.9%; *p* = 0.017 respectively), whereas autoimmune diseases were more common among females (30.6% vs. 15.6%; *p* < 0.001). Recurrence of ulcers was most common among patients with venous ulcers (*p* < 0.001) and multimorbidity among those with diabetic foot ulcers (*p* < 0.001). To conclude, males with chronic ulcers would benefit particularly from lifestyle advice, multidisciplinary treatment should be targeted specifically at those with diabetic and arterial or mixed ulcers and preventive measures at those with venous ulcers.

## 1 INTRODUCTION

Chronic ulcer patients form a heterogenous group with decidedly variable medical backgrounds, lifestyle choices and functional capacity. Ulcer aetiology likewise differs between patients; although the majority of ulcers are of vascular aetiology, namely venous‐, arterial‐ or combined arterial‐venous, that is, mixed ulcers,^1^, ^2^ diabetic foot and pressure ulcers are also relatively common.^3^, ^4^, ^5^ Evidence to date moreover shows that 10%–20% of ulcers are atypical, such as vasculitic or pyoderma gangrenosum ulcers or ulcers caused by various conditions including various inflammatory diseases, malignancies, infections, genetic disorders and systemic diseases.^6^ Furthermore, the majority of ulcer patients have a wide spectrum of different comorbidities, the most commonly reported being arterial hypertension, obesity, diabetes and dyslipidemia and also, having multiple comorbidities has shown to be common.^3^, ^7^, ^8^

For the successful treatment of ulcers, identification and treatment of the aetiological disease are of utmost importance. However, it is also crucial to recognize the comorbidities and lifestyle factors when treating patients with chronic ulcers, since these may contribute to the development and healing of an ulcer.^3^, ^9^, ^10^, ^11^ Effective treatment of ulcers is of importance to both the affected individual and to society, as chronic ulcers are associated with impaired quality of life and increased mortality and cause significant direct and indirect costs to society related to treatment, resources and loss of work and functional capacities.^12^, ^13^, ^14^, ^15^, ^16^

It is currently widely acknowledged that the most effective treatment for chronic ulcer care is achieved with a multidisciplinary approach.^17^ However, this often requires major reorganization and possibly also additional resources. Also, extensive multidisciplinary treatment is not always inevitably necessary in the treatment of all patients with chronic ulcers but should preferably be targeted at those likely to derive the greatest benefit from it. This would enable the most effective usage of available resources and would be a step towards a more personalized treatment approach. However, to facilitate this, more needs to be known about the distinctive features of different subgroups of chronic ulcer patients. Hence, this study aimed to analyse the specific characteristics of chronic ulcer patients as regard gender and ulcer aetiology using prospectively collected patients and comprehensive data primarily gathered from the validated Tampere Wound Registry (TWR).

## 2 MATERIALS AND METHODS

### 2.1 Study population and protocol

The Wound Centre of Tampere University Hospital, Finland, is a multidisciplinary tertiary care unit in charge of the treatment of patients with hard‐to‐heal chronic ulcers of various aetiologies.^18^, ^19^ Since June 2018, data have been recorded in the TWR at each visit in addition to the conventional patient records, and the TWR was recently proven to be a reliable source of clinical and research data.^19^ In this study, all 947 consecutive patients recorded in TWR during the study period 6 June 2018–31 March 2021 were initially included. However, one patient was excluded due to a completely epithelized ulcer at the first visit, and consequently, 946 patients comprised the final study cohort.

The following data recorded at the first visit to the Wound Centre were collected for the study: age, gender, current or previous smoking, mobility status and presence of comorbidities including hypertension, diabetes, hypercholesterolemia, atherosclerosis, pulmonary diseases, autoimmune diseases and previous or current cancer. Glomerular filtration rate (GFR) and body mass index (BMI) were also collected from the TWR, and if GFR was <60, the patient was determined to have kidney failure, and if the patient's BMI was >30 kg/m^2^, the patient was determined to be obese. These nine long‐term illnesses were included and also categorized into three subgroups (0–1, 2–4 and ≥5) in the analysis of total number of comorbidities. Background information related to chronic ulcers, such as history of venous thrombosis, cellulitis or erysipelas, previous vascular and ulcer related surgeries were also collected. Moreover, ulcer‐specific data including ulcer duration and size at first visit as well as most recent ulcer aetiology reported in the TWR were collected. In TWR the ulcer size was calculated with the area formula of an ellipse $\left(A=\pi \times a\times b\right)$ using the longest (a) and widest (b) ulcer measurements perpendicular to each other. In cases where ulcer aetiology, duration or ulcer measurements remained unspecified in the TWR, patients' medical records were reviewed for more specific data.

When characteristics were compared between patients with ulcers of different aetiology, aetiological subgroups of venous, arterial or mixed, diabetic foot, pressure and atypical ulcers were included. Atypical ulcers included mainly vasculitic‐ or pyoderma gangraenosum ulcers, but also other atypical ulcer types.^6^ However, patients having multiple ulcers with at least two different aetiologies (*n* = 45) and those having ulcers related to surgery or trauma or with unspecified oedema or aetiology were excluded from this analysis (*n* = 212). Finally, 689 patients were included when comparisons were made between patients with different aetiological ulcers.

The Regional Ethics Committee of Tampere University Hospital approved the study protocol (R21048R), and the study was conducted according to the guidelines of the Declaration of Helsinki as revised in 2013.

### 2.2 Statistical analyses

The data were analysed using SPSS Statistics 25.0 (IBM Corp, Armonk, NY, USA). Categorial variables were presented as numbers and percentages, and continuous variables as medians with quartiles (Q1–Q3) as the majority of the data were skewed. Characteristics of chronic ulcer patients were compared by gender and ulcer aetiology using *χ* ^2^‐test, and the Mann–Whitney *U*‐test, t‐test or Kruskal–Wallis test, as appropriate. Statistical significance was set at *p* < 0.05, and all testing was 2‐sided.

## 3 RESULTS

The whole study cohort including 946 patients had altogether 1658 ulcers. Median age of ulcer patients at their first visit to the Wound Centre was 72 (range 14–99) years, and 457 (48%) were females (Table 1). The most common comorbidities in patients with chronic ulcers were hypertension (73.3%), diabetes (49.8%), hypercholesterolemia (40.1%) and obesity (39.6%).

**TABLE 1:** Demographic, clinical and lifestyle characteristics of females and males with chronic ulcers.

	All patients		Females		Males		
	Total, *n* = 946	%/Q1–Q3	Total *n* = 457	%/Q1–Q3	Total, *n* = 489	%/Q1–Q3	*p*‐Value^a^
Age (year), median	72	61.0–81.0	76	64.0–83.5	69	58.0–78.0	**<0.001**
Hypertension	530/723	73.3	250/348	71.8	280/375	74.7	0.390
Diabetes type 1 or 2	432/868	49.8	166/419	39.6	266/449	59.2	**<0.001**
Hypercholesterolemia	266/663	40.1	107/321	33.3	159/342	46.5	**0.001**
Obesity (BMI > 30)^b^	261/659	39.6	107/298	35.9	154/361	42.7	**0.017**
Kidney failure (GFR < 60)^c^	230/668	34.4	119/312	38.1	111/356	31.2	0.059
Atherosclerosis	215/676	31.8	98/337	29.1	117/339	34.5	0.129
Autoimmune disease^d^	160/696	23.0	105/343	30.6	55/353	15.6	**<0.001**
Pulmonary disease^e^	167/772	21.6	82/377	21.8	85/395	21.5	0.938
Cancer^f^	115/687	16.7	55/335	16.4	60/352	17.0	0.826
Chronic ulcers in the past	325/738	44.0	144/346	41.6	181/392	46.2	0.214
History of cellulitis or erysipelas	213/641	33.2	96/304	31.6	117/337	34.7	0.399
Previous vascular surgery	218/706	30.9	117/340	34.4	101/366	27.6	0.050
Previous ulcer‐related surgery	141/697	20.2	62/338	18.3	79/359	22.0	0.229
History of venous thrombosis	52/610	8.5	25/297	8.4	27/313	8.6	0.926
Smoking							**<0.001**
No	429/740	58.0	240/352	68.2	189/388	48.7	
Current smoker	133/740	18.0	46/352	13.1	87/388	22.4	
Ex‐smoker	178/740	24.1	66/352	18.8	112/388	28.9	
Mobility status							**<0.001**
No walking aid	390/829	47.0	163/402	40.5	227/427	53.2	
Walks with aid	309/829	37.3	177/402	44.0	132/427	30.9	
Uses wheelchair or immobile	130/829	15.7	62/402	15.4	68/427	15.9	

*Note*: Bold indicates statistically significant difference *p* < 0.05.

Abbreviations: BMI, body mass index; GFR, glomerular filtration rate.

^a^ Females compared to males.

^b^ Body mass index >30 kg/m^2^.

^c^ Glomerular filtration rate <60 mL/min.

^d^ Rheumatoid arthritis, vasculitis, connective tissue disease, inflammatory bowel disease or other autoimmune diseases.

^e^ History of chronic obstructive pulmonary disease, asthma or other pulmonary disease.

^f^ Current or previous cancer.

When females and males with chronic ulcers were compared, males were younger at the time of the first visit (69 vs. 76 years, *p* < 0.001, Table 1). In addition, males had diabetes (59.2% vs. 39.6%; *p* < 0.001), hypercholesterolemia (46.5% vs. 33.3%; *p* = 0.001) and obesity (42.7% vs. 35.9%; *p* = 0.017) statistically significantly more often than females, whereas autoimmune diseases were more common among females (30.6% vs. 15.6%; *p* < 0.001). Compared to females, males were current or ex‐smokers more often than females (*p* < 0.001), but had better mobility status, as 53.2% of males were able to walk without aid compared to 40.5% of females. Regarding ulcer‐specific data, in females median duration of the ulcer was longer (3.7 vs. 3.4 months; *p* < 0.001) and median ulcer size larger (2.3 vs. 1.5 cm^2^; *p* < 0.001) at the time of their first visit compared to males. Median number of ulcers did not differ between genders and was one in both genders (Q1–Q3: 1.0–2.0; *p* = 0.071).

Out of the 689 patients having ulcers belonging to the aetiological subgroups studied, venous ulcers were the most commonly diagnosed in 28.5% (196 out of 689) of the patients, followed by diabetic foot ulcer (22.8%), atypical ulcer (18.7%), pressure ulcer (16.8%) and arterial or mixed ulcer (13.2%) (Table 2). Of the 129 patients with atypical ulcers, 44 patients (34.1%) had vasculitis, 33 (25.6%) pyoderma gangrenosum and 33 (25.6%) other inflammatory ulcers, such as gout, rheumatic, hidradenitis suppurativa and necrobiosis lipoidica ulcers. Further, seven patients (5.4%) had malignant ulcers, seven had (5.4%) ulcers related to infections, two had (1.6%) Martorell ulcers, two had (1.6%) radiation therapy induced ulcers and one (0.8%) livedoid vasculopathy ulcer.

**TABLE 2:** Demographic, clinical and lifestyle characteristics of chronic ulcer patients included in the main aetiological groups.

	Venous ulcer		Arterial or mixed ulcer		Diabetic foot ulcer		Pressure ulcer		Atypical ulcer^a^		
	Total, *n* = 196	%/Q1–Q3	Total, *n* = 91	%/Q1–Q3	Total, *n* = 157	%/Q1–Q3	Total, *n* = 116	%/Q1–Q3	Total, *n* = 129	%/Q1–Q3	*p*‐Value
Age (years), median	75	62–83	80	72–87	69	58–74	73	64–83	70	56–80	**<0.001**
Females	103/196	52.6	53/91	58.2	44/157	28.0	51/116	44.0	73/129	56.6	**<0.001**
Chronic ulcers in the past	98/196	50.0	40/91	44.0	66/157	42.0	23/116	19.8	34/129	26.4	**<0.001**
History of venous thrombosis	30/196	15.3	2/91	2.2	3/157	1.9	3/116	2.6	5/129	3.9	**<0.001**
History of cellulitis or erysipelas	57/196	29.1	18/91	19.8	54/157	34.4	14/116	12.1	14/129	10.9	**<0.001**
Previous vascular surgery	86/172	50.0	38/73	52.1	26/125	20.8	7/60	11.7	18/96	18.8	**<0.001**
Previous ulcer‐related surgery	16/196	8.2	18/91	19.8	36/157	22.9	19/116	16.4	11/129	8.5	**<0.001**
Smoking											0.665
No	98/164	59.8	41/77	53.2	71/130	54.6	42/73	57.5	61/101	60.4	
Current smoker	31/164	18.9	17/77	22.1	20/130	15.4	13/73	17.8	21/101	20.8	
Ex‐smoker	35/164	21.3	19/77	24.7	39/130	30.0	18/73	24.7	19/101	18.8	
Mobility status											**<0.001**
No walking aid	102/184	55.4	22/83	26.5	77/136	56.6	19/93	20.4	72/119	60.5	
Walks with aid	73/184	39.7	42/83	50.6	46/136	33.8	26/93	28.0	34/119	28.6	
Uses wheelchair or immobile	9/184	4.9	19/83	22.9	13/136	9.6	48/93	51.6	13/119	10.9	

*Note*: Bold indicates statistically significant difference *p* < 0.05.

^a^ Vasculitic, pyoderma gangraenosum, other inflammatory, malignant, postinfectious, Martorell's, radiation therapy‐induced and livedoid vasculopathy ulcers.

Gender was associated with ulcer aetiology, as males had diabetic foot (113 of 157; 72.0%) and pressure ulcers (65 out of 116; 56.0%) more often than females, whereas females were diagnosed as having arterial or mixed (53 of 91; 58.2%), atypical (73 of 129; 56.6%) and venous ulcers (103 out of 196, 52.6%) more often than males (*p* < 0.001) (Table 2). The median age of the ulcer patients differed significantly between the aetiological subgroups and patients with arterial or mixed ulcers were the oldest and patients with diabetic foot ulcers the youngest (median ages 80 vs. 69 years, Table 2).

**FIGURE 1:**
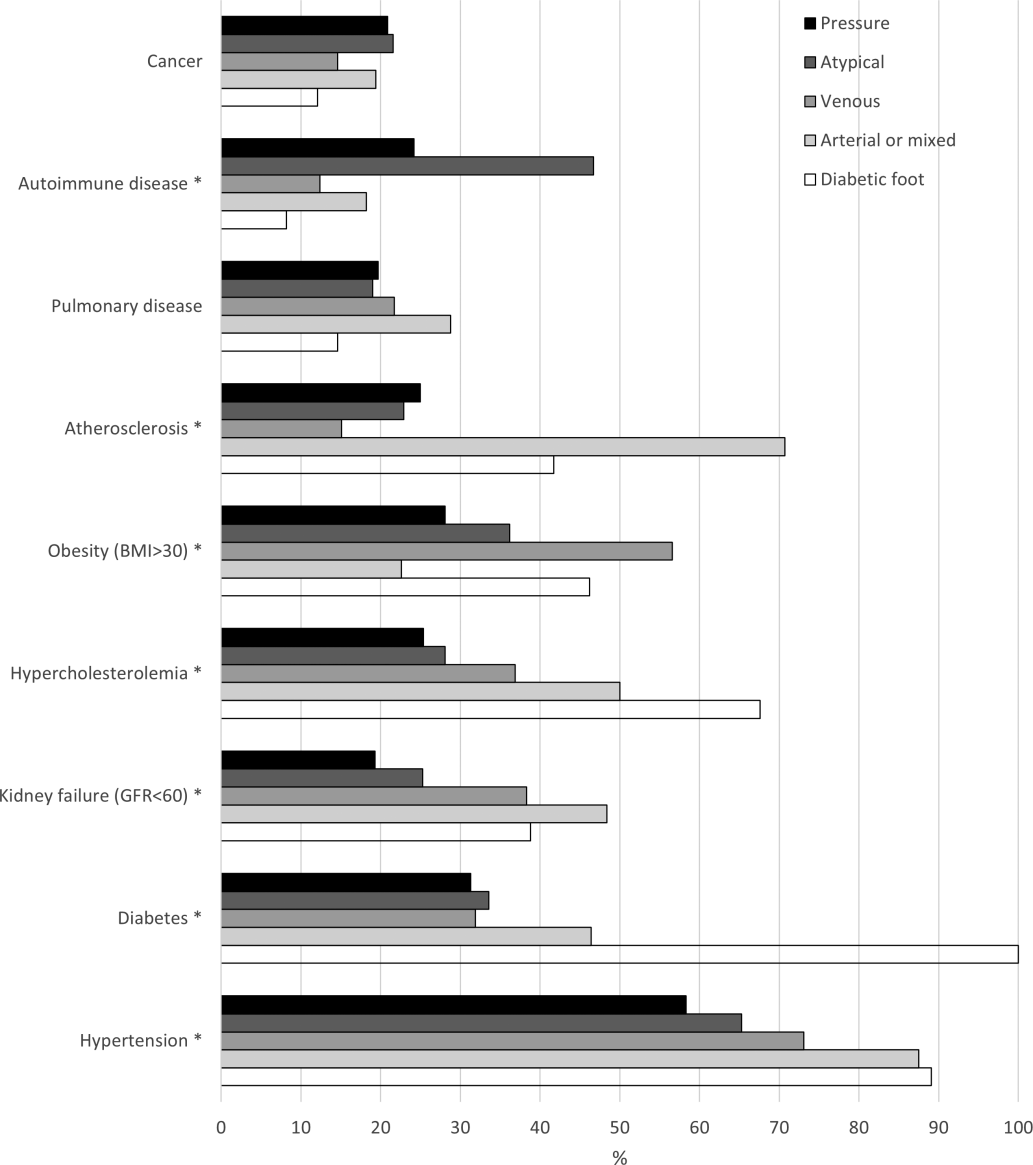
Comorbidities of chronic ulcer patients by ulcer aetiology. Only patients having one or more ulcers of the same aetiology were included in the analysis (*n* = 689); *p < 0.001.

**FIGURE 2:**
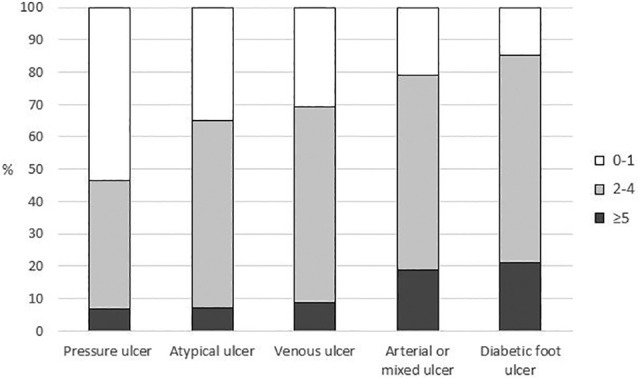
Numbers of comorbidities of chronic ulcer patients by ulcer aetiology (0–1, 2–4 and ≥5 of the following comorbidities: hypertension, diabetes, kidney failure (glomerular filtration rate <60), hypercholesterolemia, obesity (body mass index >30 kg/m^2^), atherosclerosis, pulmonary diseases, autoimmune diseases and previous or current cancer). Only patients having one or more ulcers of the same aetiology were included in the analysis (*n* = 689).

When comorbidities were compared between aetiological subgroups, statistically significant differences were detected in the prevalence of hypertension, diabetes, kidney failure, hypercholesterolemia, obesity, atherosclerosis and autoimmune diseases (*p* < 0.001 in all analyses, Figure 1). Median number out of nine comorbidities studied differed significantly between the study groups (*p* < 0.001) and was highest in patients with diabetic foot ulcer (3.0; Q1–Q3: 2.0–4.0) and lowest in patients with pressure ulcers (1.0; Q1–Q3: 0–2.0). Parallel findings were detected when comorbidities were analysed in the three previously mentioned subcategories (Figure 2). Previous chronic ulcers were most common among those with venous ulcers and erysipelas or cellulitis infections among those with diabetic foot ulcers, occurring in respectively 50.0% and 34.3% of patients (Table 2). Approximately half of the patients with venous and arterial or mixed ulcers had previously undergone vascular surgery and other ulcer surgeries were most common among those with diabetic foot ulcers. Patients with atypical ulcer had the best mobility status; 60.5% of these patients did not need walking aids compared to only 26.5% and 20.4% of those with arterial or mixed ulcer and pressure ulcer, respectively (*p* < 0.001).

Ulcer aetiology was associated with ulcer size and the median size of ulcers was greatest among patients with venous ulcer and the smallest among those with diabetic foot ulcer (median size 2.8 vs. 0.5 cm^2^; *p* < 0.001) at the time of their first visit. Median duration of an ulcer at the time of the first visit differed significantly between aetiological subgroups, and was 5.7 months in venous ulcers, 3.5 months in atypical ulcers, 3.1 months in arterial or mixed ulcers, 3.0 months in pressure ulcers and 2.6 months in diabetic foot ulcers (*p* < 0.001).

## 4 DISCUSSION

This study including a large consecutively gathered cohort of chronic ulcer patients treated in a multidisciplinary wound centre and using reliable register data established that patients with chronic ulcers are a heterogenous group of mainly elderly multimorbid individuals and that comorbidities, lifestyle and mobility status depend on both the gender of the patient and the aetiology of the ulcer. Although multidisciplinary treatment is a significant step forward in the management of chronic ulcers, this study brings a new perspective on how multidisciplinary treatment of chronic ulcers could be better planned and targeted.

In our study consisting of chronic ulcer patients with various ulcer aetiologies the most common long‐term illness was hypertension, seen in >70% of the patients, and almost half of the patients had diabetes. Further, approximately 40% had hypercholesterolemia and obesity, and one third of the patients had kidney failure. Some earlier studies on comorbidities in chronic ulcer patients report somewhat comparable prevalences of hypertension and obesity, but in our study diabetes and hypercholesterolemia were more common than previously reported.^3^, ^7^, ^8^, ^20^ It must be noted, however, that most earlier reports have mainly focused on patients with vascular ulcers,^2^, ^7^, ^8^ and a few have included diabetic and pressure ulcers,^2^, ^3^, ^20^ while studies involving patients with atypical ulcers are rare.^7^ Also, study settings have differed as, for example, in a fairly small Swedish study patients were invited by their respective municipalities by postal questionnaire,^2^ whereas in most studies patients were treated in specialized wound centres or leg ulcer clinics.^3^, ^7^, ^8^ Moreover, compared to some studies with lower diabetes prevalence,^3^, ^7^ our study cohort was gathered almost a decade later and the incidence of diabetes has been shown to increase worldwide^21^ which at least partly explains this difference. Thus, this study emphasizes that diabetes is an increasingly important comorbidity in patients with chronic ulcers of all aetiologies, and therefore screening for diabetes should be carried out in all patients with chronic ulcers.

There was a slight predominance of males in our study cohort, which contradicts most,^2^, ^3^, ^7^, ^8^ but not all, earlier studies.^20^ Males, as this study established, more often had metabolic diseases, such as diabetes, obesity and hypercholesterolemia than females, and further, smoking was associated with male gender. This highlights the necessity of nutritional and lifestyle guidance particularly for males with chronic ulcers. Instead, autoimmune diseases were twice as common in females and as several autoimmune diseases predispose to vasculitis,^22^ atypical ulcers were likewise fairly consistently more common in females than in males. Furthermore, among our consecutive hospital‐treated ulcer patients females were older and had poorer mobility status than males. Hence, physiotherapy and assessment of aids needed in daily activities and self‐care are imperative, especially for females. Rather interestingly, the durations and sizes of ulcers were longer and larger in females than in males, and as both are known risk factors for poor ulcer healing,^23^, ^24^ this may imply that females in particular are at risk of delayed ulcer healing. However, this finding was fairly logical as ulcer aetiologies are more common among males, particularly diabetic foot ulcers, are often smaller and also more promptly referred to tertiary care than, for example, venous ulcers predominating in females, findings are also apparent in the present study.

In the present study venous ulcers were associated with well‐known risk factors for venous ulcers, such as female gender,^25^, ^26^ history of venous thrombosis,^25^, ^27^ erysipelas or cellulitis of the lower extremities,^25^ previous vascular surgeries^25^ and obesity.^26^, ^27^, ^28^ Moreover, previous chronic ulcers were particularly common in those with venous ulcers in this study and also reported by others.^27^, ^29^ Therefore it is of utmost importance to minimize the risk of ulcer recurrence by targeting all possible risk factors. Effective interventions for the prevention of recurrent erysipelas and cellulitis have been shown to be not only antibiotics,^30^ but also compression therapy.^31^ Also, dietary counselling by a nutritionist and weight management are essential, especially as previous venous ulcer patients have also been reported to be prone to recurrences if treatment for obesity is not successful.^28^

In addition to traditional risk factors for peripheral arterial disease, such as increased age, hypercholesterolemia, atherosclerosis and diabetes,^10^ in this study almost half of the chronic ulcer patients with arterial or mixed aetiology had kidney failure. Chronic kidney failure has been recognized as a risk factor for peripheral arterial disease, but awareness of its critical importance as a risk factor for arterial ulcers and even leg amputations is growing^32^ and also supported by this study. In addition to pressure ulcers, poorer mobility status was associated with arterial or mixed ulcers, but this is most likely largely explained by the higher age of this patient group. Multimorbidity was associated with arterial or mixed ulcers, but to an even greater extent with diabetic foot ulcers. These patients had diseases requiring multimodal treatment, and can therefore benefit from ulcer‐specialized multidisciplinary treatment most readily organized in a wound centre. Conversely, such resource‐consuming treatment does not seem to be as essential among those with pressure ulcers and only few comorbidities.

Rather alarmingly, median ulcer durations prior to first wound centre visit were relatively long, ranging from 2.6 to 5.7 months in this study. Prompt referral is especially needed for those patients with peripheral arterial disease, diabetic foot ulcer and atypical ulcers such as vasculitis or pyoderma gangrenosum. Delayed referral increases the risk not only of delayed ulcer healing but also risks of severe complications, such as infections and leg amputations.^33^, ^34^ Hence high‐level knowledge in specialized wound centres alone is not adequate; heightened awareness in primary health care of diagnostics, treatment as well as referral policies for chronic ulcers is crucial, and education should additionally be directed at general practitioners, often the first to encounter ulcer patients. Also, informing patients in risk groups, such as those with diabetes, about not only prevention but also about alarming symptoms and signs might shorten the patient‐dependent diagnostic delay.

The major strengths of our study were the large number of consecutive patients recorded in a validated registry designed specifically for the reliable documentation of chronic ulcer patients.^19^ Moreover, a wide spectrum of ulcer aetiologies also including atypical and diabetic foot ulcers were included and comprehensive data on comorbidities, lifestyle and other risk factors as well as ulcer‐specific data were analysed. However, the present study included mostly hard‐to‐heal ulcers treated at a multidisciplinary tertiary care unit, and therefore the results may not reflect all ulcer patients treated, for example, in primary health care where the prevalences of various ulcer aetiologies and ulcer severities and patients' health conditions overall might differ quite substantially from our patients. Also, although we comprehensively analysed prevalences of different comorbidities, we were unable to evaluate disease severities, which could constitute a limitation. Further, as this was a retrospective registry study, only parameters gathered in routine patient care in our unit were included, and, for example, patient perspective was not investigated in detail nor did we have exact ulcer areas available but instead, ulcer lengths and widths. We also emphasize that in this study no adjustments for age and gender were performed as our aim was to characterize different subgroups of ulcer patients as is, that is, as they are encountered in treatment units since this ‘real‐world’ information is most valuable when reorganizing resources and patient care.

In conclusion, the findings of the present study confirmed that chronic ulcer patients are a heterogenous patient group and more precise characterization of subgroups is needed and also feasible. This study established that gender and ulcer aetiology are factors associated with different comorbidities and risk factors for ulcers. Future studies are needed, however, to further identify specific characteristics of patients with various types of chronic ulcers to enable more efficient individualized management. Nevertheless, as most ulcer patients in the present study had comorbidities and a significant portion had poor lifestyle choices or restricted mobility, the need for multidisciplinary treatment and a holistic approach in general was reinforced and furthermore, individualized assessment and treatment is essential.

## FUNDING INFORMATION

This study was financially supported by the Tays Project Nos. 9X061, 9AC033, MK326 and MJ006M.

## CONFLICT OF INTEREST STATEMENT

The authors have no conflicts of interest to declare.

## Data Availability

The data that support the findings of this study are available on request from the corresponding author. The data are not publicly available due to privacy or ethical restrictions.
